# Embryonic and Larval Development of Stinging Catfish, *Heteropneustes fossilis*, in Relation to Climatic and Water Quality Parameters

**DOI:** 10.3390/life13020583

**Published:** 2023-02-19

**Authors:** Balaram Mahalder, Mohammad Mahfujul Haque, Mohammad Abu Baker Siddique, Neaz A. Hasan, Md. Mehedi Alam, Md. Mahamudun Naby Talukdar, Mobin Hossain Shohan, Nusaifa Ahasan, Md. Mahmudul Hasan, A. K. Shakur Ahammad

**Affiliations:** 1 Department of Aquaculture, Faculty of Fisheries, Bangladesh Agricultural University, Mymensingh 2202, Bangladesh; 2 Department of Fisheries Biology and Genetics, Faculty of Fisheries, Bangladesh Agricultural University, Mymensingh 2202, Bangladesh; 3 Department of Fisheries and Marine Bioscience, Bangabandhu Sheikh Mujibur Rahman Science and Technology University, Gopalganj 8100, Bangladesh; 4 Department of Fishery Resources Conservation and Management, Faculty of Fisheries and Ocean Sciences, Khulna Agricultural University, Khulna 9100, Bangladesh

**Keywords:** *Heteropneustes fossilis*, embryo, larval development, climatic variables, water quality parameters, Bangladesh

## Abstract

In terms of hatchery-based seed production, one of the most important aquaculture species in Bangladesh is the stinging catfish (*Heteropneustes fossilis*). Scientific and evidence-based embryonic and larval development research on this fish species in the context of climate change is limited. This experimental study was conducted via induced breeding of stinging catfish using a conventional hatchery system, rearing the larvae in hapas placed in ponds. A series of microscopic observations using a trinocular digital microscope and an analysis of the relationship between larval growth and climate-driven water quality parameters such as temperature, pH, dissolved oxygen, total dissolved solids, alkalinity, and ammonia were performed. During embryonic development, the first cleavage was observed between 30 and 35 min of post-fertilization. Embryonic development (ranging from the 2-cell to the pre-hatching stage) took 21:00 h. Hatching occurred at 22:30 to 23:00 h after fertilization, with an average larvae length of 2.78 ± 0.04 mm. In the post-hatching stage, four pairs of tiny barbels appeared at 36:00 h, and the larvae started feeding exogenously after 72:00 h. These larvae fully absorbed their yolk sacs on the 6th day and attained an average length of 6.44 ± 0.06 mm. Aerial respiration of the larvae was investigated through naked-eye observation on the 10th day of hatching. The average length of the larvae was 32.00 ± 2.0 mm at the end of the 30-day post-hatching observation period. Bivariate correlation analysis showed significant correlations between key climatic variables and water quality parameters under hapa-based larval-rearing conditions. According to canonical correlation analysis, the first canonical function revealed the highest significant correlation between the two sets of variables (r1 = 0.791). The response variable weight of larvae (6.607) was linked to two explanatory variables: pH (0.321) and dissolved oxygen (0.265). For the second canonical correlation function, a positive correlation (0.431) was observed between the two sets of variables. Larval weight (−18.304) was observed to be linked to climatic variables, including air temperature (−0.316) and surface pressure (0.338). Results of this study reveal the subtle correlation between larval growth and water quality driven by climatic variables.

## 1. Introduction

One of the most fundamental challenges for the study of the developmental biology of fish is the standardization of embryonic and larval development [1]. Therefore, staging a series of embryonic and larval development is a useful standardization tool that provides accuracy for developmental studies. Aquaculture, the fastest growing [2] driver of fish production in Bangladesh, strongly depends on hatchery-based fish seed production. In the aquaculture industry, that is, hatchery-based fish seed production, the seed production of new species is increasing. One of the problems that hatchery operators face in hatchery-based fish fry production is a lack of technical knowledge on how to rear broodfish, carry out breeding operations, rear larvae, feed larvae, and so forth. Because larval development occurs in a very short period of time, changes in the larval stage during rearing are very sensitive to ambient water quality parameters and weather parameters, and cannot be seen with the naked eye, there is a great dearth of operator knowledge. Due to the lack of such technical knowledge and skills, hatchery operators cannot obtain the desired yield of fish fry from their hatcheries. Furthermore, the quality of the fry produced remains low, and the mortality rates are high since hatchery operators suffer financially [3,4]. Therefore, aquaculture farmers face huge financial losses owing to the use of poor-quality fingerlings for fish farming.

The stinging catfish or Indian catfish *Heteropneustes fossilis* (Bloch), locally known as shing or shingi, is a popular and intensively used commercial aquaculture species in Bangladesh [5]. According to the tradition of Bengali culture, the species is considered a nutritional supplement for convalescing patients because of its unique taste, high nutritional value, low fat, high iron content, and medicinal properties [6]. The demand for stinging catfish culture has increased significantly in Bangladesh, but natural or wild sources of fry are insufficient to meet the demands of commercial aquaculture farms [7]. Moreover, there is a negative perception at the fish farmer level regarding the quality of hatchery-produced stinging catfish fry and fingerlings [8]. Hatchery-produced hatchlings of stinging catfish have been reported to have a high mortality rate, and most of those that survive are deformed [9,10,11]. For ensuring a consistent supply of quality fingerlings to meet the demands of aquaculture farmers, techniques for induced breeding, embryonic development, and larval rearing must be developed. Hatchery operators urgently need clear knowledge and skills, especially with regard to larval rearing [12].

As hatchery operators lack biological knowledge of embryonic and larval development, they also lack sufficient knowledge of how different climatic and water quality parameters affect embryonic and larval development. Climatic variables, such as temperature and rainfall, and changes in water quality parameters, such as dissolved oxygen (DO), pH, and alkalinity, significantly affect embryonic and larval growth [13,14,15]. According to a recent study [16], fish hatcheries including hatcheries of stinging catfish are being negatively affected by various climatic changes, such as changes in air and water temperatures, rainfall, and sunlight intensity, as well as frequent natural disasters, resulting in disruption of hatchery production with poor quality fry. Among the different climatic and water quality parameters, temperature is a fundamental factor that influences other parameters in various ways to affect broodfish reproductive processes, such as growth, maturation, ovulation, breeding, embryonic development, hatching of eggs, and larval and juvenile growth and survival [17]. In addition to temperature effects, fish embryonic and larval development are critically affected by pH, DO, salinity, ammonia, and other climatic factors [18,19,20,21]. Fish embryos and larvae are more sensitive to changes in pH than are juveniles and adults [22]. In addition to biological factors, physical and chemical parameters also affect egg development [23]. Understanding the biology, nutritional needs, and environmental preferences of a specific species requires knowledge of its embryonic and larval development. Owing to limitations in the advancement of science in the early nineties, particularly with respect to mediocre microscopy equipment, it has been challenging for researchers to study the larval development of fishes. Despite these scientific limitations, a few studies have been conducted on the reproductive biology of stinging catfish [24,25]. No comprehensive studies have been conducted on embryonic and larval development in relation to climatic and water quality parameters through microscopic observation at different larval stages for stinging catfish, except for a very old study [26] performed via traditional microscopic observation. Although fish ontogeny science has advanced over time, certain constraints on egg development are still unknown [23].

The development of high-resolution digital microscopes from analog microscopes, including image-processing utilities, which allows digital microscope users to capture, store, and process digital images of an object, is being reported in the scientific literature [27]. Digital microscopes and their image-processing systems are largely capable of enhancing observed biological objects and performing other operations according to user needs.

The issues evident from the above discussion indicate that there are limited studies on the morphological and chronological developmental stages of stinging catfish larvae, along with the fact that hatchery operators do not have a clear biological knowledge of the various stages of larval development. The purpose of this study is to reveal the embryonic and larval developmental stages of stinging catfish, express the morphological and chronological developmental stages in image form, and understand how climatic and water quality parameters affect larval growth in prevailing uncontrolled environmental conditions.

## 2. Materials and Methods

### 2.1. Ethical Statement

This study included samples of fish from the pond as well as those bred in captivity. All animal scientific procedures were rigorously followed with prior approval by the Animal Welfare and Ethics Committee of the Bangladesh Agricultural University (BAU) (Ref. no. BAURES/ESRC/FISH-11/2022).

### 2.2. Study Location

The investigation of breeding and embryonic development of stinging catfish was conducted in the hatchery unit of the Intensive Aquaculture Systems Laboratory (24°43′30.61″ N, 90°26′4″ E), which belongs to the Department of Aquaculture and is situated near the Faculty of Fisheries, BAU, Mymensingh. The brood stock and larval development of stinging catfish were conducted in the earthen pond and the hapa (A hapa is a cage-like, rectangular, or square net impoundment placed in a pond for holding fish for various purposes) in the pond, respectively, located in front of the Intensive Aquaculture Systems Laboratory.

### 2.3. Collection and Rearing of Stinging Catfish Broodstock in the Earthen Pond

Stinging catfish broodstocks were acquired from a renowned fish farm, Anil Matshay Hatchery, located in Kewatkhali, Mymensingh Sadar, Mymensingh, Bangladesh. Male (average total length and live weight of 14.44 ± 0.17 cm and 16.46 ± 0.42 g, respectively) and female (average length and live weight of 16.12 ± 0.18 cm and 22.48 ± 0.85 g, respectively) broods of stinging catfish were stocked in an earthen pond (23.78 × 10.06 × 1.5 m^3^) equipped with inlet–outlet and water exchange facilities in early January 2022. The broodstocks were reared and domesticated for 6 months by providing feed twice daily with 35% protein-containing commercial floating feed (brand name: Mega Feed) at a rate of 3–4% of body weight.

### 2.4. Selection and Acclimatization of the Stinging Catfish Broodstock

Mature male and female broodfishes were collected from the broodstocks’ pond and differentiated by secondary sexual characteristics. The mature males had flat abdomens and long and pointed genital papillae, and the mature females had a soft, large, swollen abdomen and a round swollen genital opening. A collection of the broods, male and female, was transferred to separate hatchery tanks for acclimatization with continuous showering of water to increase their reproduction response. Continuous aeration was provided to all tanks.

### 2.5. Induced Breeding of Stinging Catfish 

For this experiment, five females and ten males were collected from broodstocks’ pond for artificial breeding using Pituitary Gland Extracts (PGE). The females (average total length and live weight of 23.15 ± 0.37 cm and 81.08 ± 2.95 g, respectively) were treated with a single dose of PGE at 75 mg kg^−1^ body weight [28] while the males (average length and live weight of 17.9 ± 0.38 cm and 28.0 ± 2.01 g, respectively) were treated with 10 mg kg^−1^ body weight [7]. Acetone-dried pituitary glands were macerated in a tissue homogenizer by adding a measured quantity of distilled water. The extract was then centrifuged at 2000 rpm for 10 min, and the supernatant fluid was drawn into a syringe for injection. Hormones were administered to the fish bodies through injection. After injecting the PG extract, female and male fish were released into two hapas separately, which were placed in the hatchery cistern. In the hapas, continuous water showering was maintained to stimulate the broodfish. Nine hours after the injections, the broods were removed, after ovulation was complete. The females were stripped and the eggs were kept in a bowl. The males were sacrificed to remove the testes. The testes were then cut into pieces inside a Petri dish, and the milt was diluted with physiological saline (0.9% NaCl) at 1:3.5 ratio. The eggs and diluted spermatozoa were mixed with sufficient water in the bowl to fertilize the eggs. The eggs were placed into three hapas (size of the each hapa was 100 × 100 × 60 cm^3^), each placed in separate breeding tanks. Photographs of embryonic development at each stage were taken until the eggs were hatched. The time of occurrence of each developmental stage was recorded.

### 2.6. Determination of the Breeding Performance of Stinging Catfish

To determine the fertilization rate, 100 eggs were randomly taken from each replicate and placed in a Petri dish. Fertilized eggs that appeared blackish or greenish in color were counted. Subsequently, to observe hatching rates, 100 fertilized eggs were separated into three hatching bowls provided with oxygen and water flow from the three hapas. The total number of hatchlings was counted after hatching. The fertilization and hatching rates were calculated using the following formula:

(i) Fertilization rate (%) = (no. of fertilized eggs/total no. of eggs) × 100
(ii) Hatching rate (%) = (no. of eggs hatched/total no. of fertilized eggs) × 100

### 2.7. Egg Incubation and Image Analysis of Embryonic and Larval Development of Stinging Catfish

To examine embryonic development, 10–15 eggs were randomly taken from the hapas every 30–60 min until hatching. Different stages of embryonic development were observed on a computer monitor using an Olympus CX 43 biological microscope attached to an Olympus EP50 microscope camera at 40× magnification. Photographs of embryonic development and different parts of the larvae were captured using the microscope-attached camera. The developing embryos were measured using the image-analyzing software EP view (Olympus Corporation, Tokyo, Japan). Larval development was observed every 4 h for the next 3 days. On the fourth day, 500 larvae were transferred into each of three hapas (1 m^3^ each) placed in the pond. Larvae were fed egg yolk from the fourth day for 1 week, and then fed nursery powder containing 45% protein four times per day from the 11th day. Larval length and weight were measured once daily for 30 days. Larval weight was measured using an electronic precision balance (FSH, A&D Store, Wood Dale, IL, USA) at a division range of 0.001 g. Larvae photographs were taken with a scale of known length using a DMWifi microscope (android-based portable wireless digital microscope) and, finally the length was calculated using “ImageJ” software, which is a Java-based image-processing program. Before that larval photographs were taken under live conditions, and the data were recorded as mean ± SE.

### 2.8. Determination of Climatic Variables and Water Quality Parameters

Data regarding climatic variables, such as air temperature, humidity, and surface pressure were collected from the local weather station at Bangladesh Agricultural University, Mymensingh. The major water quality parameters were recorded daily. Water quality parameters, such as water temperature, DO, pH, Total Dissolved Solids (TDS), and ammonia, were measured daily using a SMART sensor temperature meter (SMART Sensor AR 867), DO meter (Lutron DO-5509), pocket-sized pH meter (pH-107), TDS meter (TDS-3 Pen portable TDS meter), and ammonia test kit, respectively, during the study period.

### 2.9. Data Analysis

The data generated from this experimental study were recorded in MS Excel 2016, and descriptive statistical analyses, particularly focusing on the mean and standard deviation of the timing and growth of embryos and larvae of stinging catfish, were performed using SPSS (Statistical Package for Social Science) version 23 (IBM SPSS Statistics 23). Canonical correlation analysis (CCA) was conducted using RStudio (4.2.2) to determine the relationship between larval development and climatic and water quality parameters. The first step of CCA is to derive one or more canonical functions. Each function consists of a pair of variables, one as an explanatory or independent variable, such as water quality parameters (DO, pH, water temperature, and TDS), or climatic variables (air temperature, humidity, and surface pressure), and growth parameters (length and weight) as response or dependent variables. The first function to be extracted accounts for the maximum amount of variance in the two sets of variables, that is, the first pair of canonical variates exhibits the highest inter-correlation possible between the two sets of variables. The second pair of canonical variates accounts for the maximum amount of the remaining variance. Typically, two criteria are used for selecting the canonical function for interpretation: the level of statistical significance of the function and the magnitude of the canonical correlation. In this study, explanatory variables were described by *Y* variables in vector *c* = (*c*_1_, *c*_2_,…, *c*_Y_), and response variables were described by *X* variables in vector *k* = (*k*_1_, *k*_2_,…, *k*_*X*_). Thus, *n* number of observations *j* can be described by the vectors:

$$\begin{array}{l} \left[\begin{array}{c} c_{1}\\ k_{1} \end{array}\right]\ldots \ldots \left[\begin{array}{c} c_{n}\\ k_{n} \end{array}\right] \end{array}$$

with a partitioned sample

$$\begin{array}{l} \bar{x}=\left[\begin{array}{c} {\bar{x}}_{C}\\ {\bar{x}}_{k} \end{array}\right] \end{array}$$

and a partitioned sample variance

$$\begin{array}{l} S=\left[\begin{array}{cc} S_{cc} & s_{kc}\\ S_{kc} & S_{kk} \end{array}\right] \end{array}$$

where

$$\begin{array}{l} S_{cc}=\frac{1}{n-1}\sum_{j=1}^{n}\left(c_{j}-{\bar{x}}_{c}\right){\left(c_{j}-{\bar{x}}_{c}\right)}^{\prime } \end{array}$$

$$\begin{array}{l} S_{kk}=\frac{1}{n-1}\sum_{j=1}^{n}\left(k_{j}-{\bar{x}}_{k}\right)\left(k_{j}-{\bar{x}}_{k}\right)\prime \end{array}$$

and

$$\begin{array}{l} S_{ck}=\frac{1}{n-1}\sum_{j=1}^{n}\left(c_{j}-{\bar{x}}_{c}\right)\left(k_{j}-{\bar{x}}_{k}\right)\prime \end{array}$$

The variance–covariance matrices *S_cc_* and *S_kk_* include the variances and covariance within groups for the explanatory and response variables, respectively. The variance–covariance matrices *S_kc_* and *S_ck_* include the covariance between the variables from different groups. The linear combination of the explanatory and response variables results in the respective sets of canonical functions *s* = (*s*_1_, *s*_2_, …, *s_Y_*) and *p* = (*p*_1_, *p*_2_, …, *p_X_*). The vectors include climatic variables (explanatory variables) in canonical variates, and it results from the linear combination of the *c* vector and the canonical coefficients vector *a*, as *s* = *a*’*c*. Vector *p* contains the growth parameters (response variables) under canonical variates, and it results from the linear combination of the vector *k* and the canonical coefficient vector *b*, as *p* = *b*’*k*. The canonical correlation coefficients *a* and *b* were estimated using the variance–covariance matrices of the original sets of explanatory and response variables, as explained by Equations (1) and (2), respectively:

(1) $$\begin{array}{l} S_{cc}^{-1}S_{ck}S_{kk}^{-1}S_{kc}a_{i}=\lambda_{i}a_{i} \end{array}$$

(2) $$\begin{array}{l} S_{cc}^{-1}S_{ck}S_{kk}^{-1}S_{kc}b_{i}=\lambda_{i}b_{i} \end{array}$$

Pairs of canonical variables (*s_i_*, *p_i_*) produced by the linear combination of *s_i_* = *a_i_*’*c* and *p_i_* = *b_i_*’*k* have greater correlation and are more independent than the pairs of variables (*s_i_* + 1, *p_i_* + 1) produced by *s_i_* + 1 = *a_i_* + 1’*c* and *pi* + 1 = *b_i_* + 1’*k*. This process was repeated until the last pairs of canonical variables, *s_Y_* and *p_Y_*, were obtained. Thus, CCA maximizes the correlation between canonical variable pairs *s*_1_ = *a*_1_’*c* and *p*_1_ = *b*_1_’*k*. The second pair of canonical variables presents the second largest correlation, *s*_2_ = *a*_2_’*c* and *p*_2_ = *b*_2_’*k*, uncorrelated with the *s*_1_ and *p*_1_ values.

## 3. Results

### 3.1. Climatic Variables and Water Quality Parameters during the Study

The observed climatic parameters such as air temperature, relative humidity, air pressure and water quality parameters, such as pH, DO, TDS, and ammonia, had no critical values. Water temperature variation from 27 to 31 °C was observed in the hapas (Table 1). The pH values ranged from 7.4 to 8.6, and the DO ranged from 4.9 to 11.81 ppm.

### 3.2. Fertilization, Hatching, and Embryonic Development of Stinging Catfish

**Figure 1:**
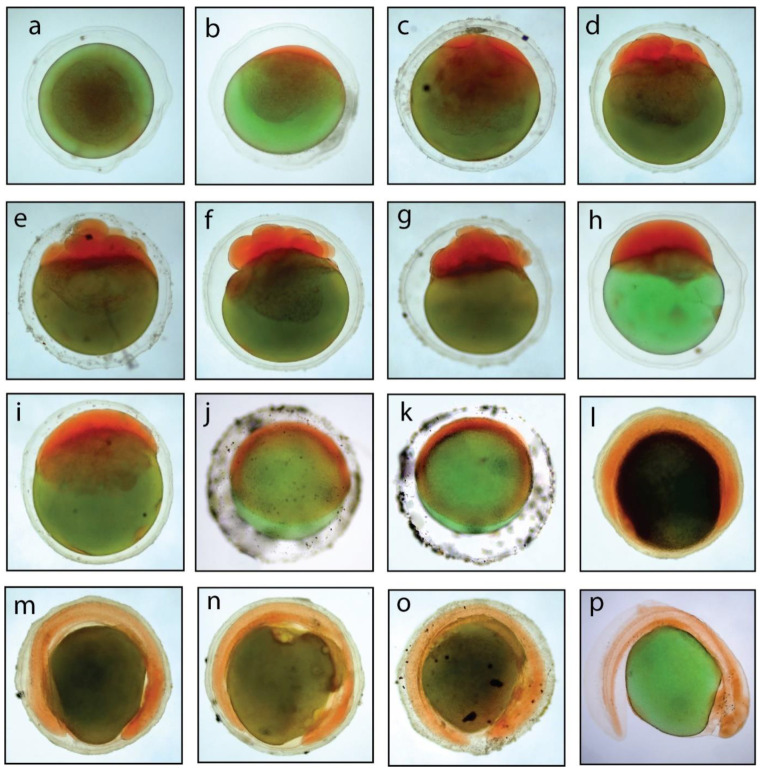
Embryonic development of stinging catfish. (**a**) Fertilized egg; (**b**) Blastodisc formation; (**c**) 2-cell stage; (**d**) 4-cell stage; (**e**) 8-cell stage; (**f**) 16-cell stage; (**g**) 32-cell stage; (**h**) 64-cell stage; (**i**) Morula stage; (**j**) Blastula stage; (**k**) Gastrula stage; (**l**) Somatic formation; (**m**) Yolk plug; (**n**) Twisting movement; (**o**) Pre-hatching; (**p**) Newly hatched larvae.

The fertilization and hatching rates of stinging catfish in this study were 68% and 69%, respectively. Following egg fertilization, the zygote was formed, and embryonic development began, culminating in hatching. The embryonic developmental stages of stinging catfish immediately after fertilization are summarized in Table 2 and Figure 1. The fertilized eggs were transparent, spherical in shape, reddish green, and demersal, with an average diameter of 1355.06 ± 17.15 μm. The egg yolk was greenish, and the embryonic disc was laterally displaced to the sides. The egg membrane was separated from the yolk by using a small perivitelline space (Figure 1a). The reddish blastodisc at the pole of the fertilized egg could be easily identified with the naked eye. The blastodisc formed 20 min after fertilization, was reddish in color, and appeared at the animal pole (Figure 1b). The egg diameter after blastodisc formation was measured as 1364.88 ± 28.43 μm. The first cleavage that split the blastodisc into two parts occurred within 30–35 min of post-fertilization (Figure 1c) with a diameter of 1438.09 ± 25.29 μm. The second cleavage (4-cell stage) appeared 40 min after fertilization with a diameter of 1440.59 ± 47.69 μm. The third division (8-cell stage) was observed between 1:00–1:10 h of post-fertilization, with a diameter of 1457.19 ± 9.92 μm (Figure 1e). The 16-cell, 32-cell, and multi-cell (64-cell) stages were observed at 1:20 h, 2:00 h, and 2:30 h of post-fertilization, respectively, with egg diameters of 1472.02 ± 13.17 μm, 1472.16 ± 23.46 μm, and 1475.81 ± 19.64 μm (Figure 1f–h), respectively. The size of the blastomeres reduced with continuous and successive cleavage and attained the morula stage at 2:50–3:00 h after fertilization with an average diameter of 1480.44 ± 30.88 μm (Figure 1i). The blastula stage appeared at approximately 4:00–4:15 h post-fertilization, and was characterized by a flattening of the cellular material and the formation of a blastodisc with numerous blastomere cells over the yolk sac. The mean diameter was 1486.01 ± 1.22 μm (Figure 1j). The gastrula stage started at 6:35 h post-fertilization with a mean diameter of 1491.03 ± 30.56 μm and marked the onset of epiboly (Figure 1k). The blastoderm was further spread on both sides and flattened at the top, resulting in the formation of the germinal ring. Somatic formation started at 9:00 h post-fertilization, which continued up to 25 somites by 18:00 h post-fertilization, with an initial diameter of 1509.26 ± 40.58 μm (Figure 1l). After 19:00 h post-fertilization, the yolk plug formed with a resulting diameter of 1523.80 ± 31.66 μm (Figure 1m). The twisting movement of the embryo in the egg was detected at approximately 20:00–21:00 h after fertilization, with an egg diameter of 1551.90 ± 23.59 μm (Figure 1n). Pre-hatching started at 21:00 h post-fertilization with a diameter of 1647.44 ± 40.61 μm (Figure 1o). Hatching occurred at 22:30 h after fertilization (Figure 1p).

### 3.3. Larval and Post-Larval Development of Stinging Catfish

**Figure 2:**
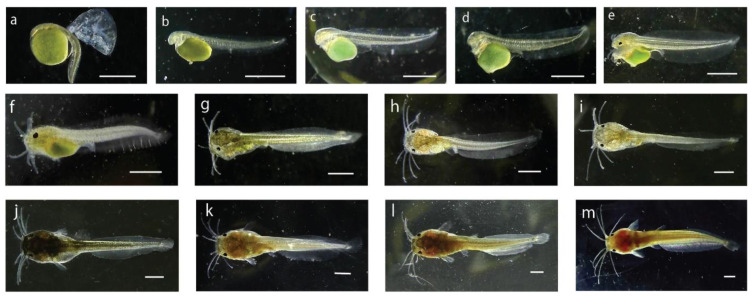
Larval development of stinging catfish, (**a**) Hatchling; (**b**) 4 h old larva; (**c**) 8 h old larva; (**d**) 24 h old larva; (**e**) 36 h old larva; (**f**) 48 h old larva; (**g**) 3 d old larva; (**h**) 6 d old larva; (**i**) 10 d old larva; (**j**) 15 d old larva; (**k**) 20 d old post-larva; (**l**) 25 d old post-larva (**m**) 30 d old post-larva.

**Figure 3:**
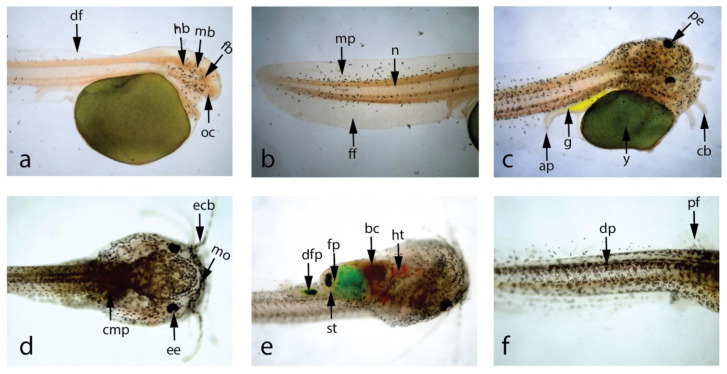
Photomicrographs showing larval development of stinging catfish, (40×). (**a**) Newly hatched larva with oval-shaped yolk sac, (**b**) Posterior end of newly hatched larva, (**c**) 36 h larva with pigmented eye, (**d**) Dorsal view of 9 d larva, (**e**) Ventral view of 9 d larva, (**f**) Dense pigmentation on 11 d larva body, (ap—anal pore, cb—chin barbels, cmp—concentrated melanophores, df—dorsal fin, dfp—digested food particles, dp—dense pigmentation, ecb—elongated chin barbells, ee—epicanthus of eye, fb—fore brain, ff- fin fold, fp—food particles, g—gut, hb—hind brain, ht—heart, mb—mid brain, mo—mouth, mp—melanophores, oc—optic cup, pe—pigmented eye, pf—pectoral fin bud, st—stomach, y—yolk sac.

The larval stage of a fish is a vital period in its life cycle due to its delicate ontogenetic development, which includes significant changes in body tissue structure, organ development, and system development. The larval stage, which begins with an endogenous source of yolk reserves and progresses to extrinsic feeding, is also crucial for nutrition. As substantial fish mortality occurs in aquaculture during the larval stage, understanding the structural and functional changes of the larval digestive system is critical for enhanced fish survival during larval rearing. This study analyzed larval development using photomicrographs of the morphological development of newly hatched stinging catfish larvae. The detailed changes in morphometry of stinging catfish from the day of hatching to the 30th day of life are presented in Figure 2 and Figure 3 and Table 3. At the age of hatching (22:30 h), the average length and weight of the larvae were 2.78 ± 0.04 mm and 0.002 g, respectively. At 36 h of age, eye pigments appeared. The yolk sac gradually shrunk and became exhausted at 6th day (Figure 2h). In terms of morphological changes after hatching, the larvae had large yolk sacs, which were green in color. From the fourth day after hatching, the larvae were fed with egg yolk for 10 days. From the 11th day, they were fed with 45% protein-containing powdered feed until 30 days post-hatching. Later, larvae of stinging catfish underwent various physical changes, such as fin growth, barbless lengthening, and visible lateral spines, thus transforming into full-fledged stinging catfish after 30 days. All of these morphological changes were accompanied by color changes in the larval body (Figure 3a–f).

### 3.4. Correlation between Larval Development and Climate-Driven Water Quality Parameters

Bivariate correlation analysis showed that there were significant relationships between key climatic and water quality parameters in hapa-based larval rearing conditions (Table 4). A significant (*p* < 0.05) positive correlation was revealed between air and water temperatures. A significant negative correlation was observed among air temperature, humidity, dissolved oxygen, and TDS. For the water temperature, a negative correlation was observed with the dissolved oxygen content. These results show that climatic parameters, particularly air temperature, have direct impacts on various water quality parameters on which larval growth depends.

To explore the correlations between multivariate sets of variables, canonical correlation analysis (CCA) was performed to exhibit canonical functions, as summarized in Table 5. CCA depicts the pattern of correlations between climatic variables (air temperature, surface pressure and humidity) and water quality parameters (water temperature, DO, pH, and TDS) in relation to embryonic and larval growth parameters (length and weight) under hatchery conditions and in hapas set in the pond. In this analysis, climatic variables and water quality parameters were explanatory or independent variables, denoted by Y, while the embryonic and larval growth parameters were responsive or dependent variables, denoted by X. Estimated canonical correlations between the pairs of canonical variates were revealed to be 0.791 and 0.431 with their probabilities of significance being 0.013 and 0.055, respectively (Table 5).

### 3.5. Characteristics of Canonical Function 1

**Figure 4:**
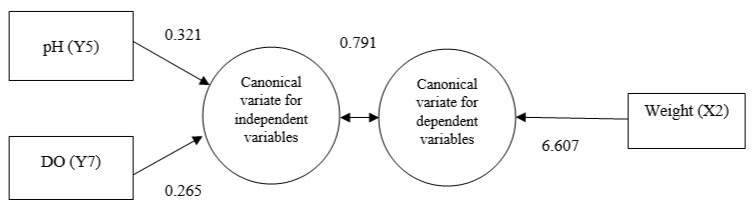
Canonical function 1 (adapted from Table 5).

The first canonical function reflected a strong correlation (0.791%) between water quality and growth parameters (Figure 4). Under this function, the responsive or dependent variable, the weight, achieved a high canonical loading (6.607) that collectively explained 73.5% of the variance, while the explanatory DO loaded strongly (0.265) together with the loading (0.321) of pH and collectively explained 33.6% of the variance.

### 3.6. Characteristics of Canonical Function 2

**Figure 5:**
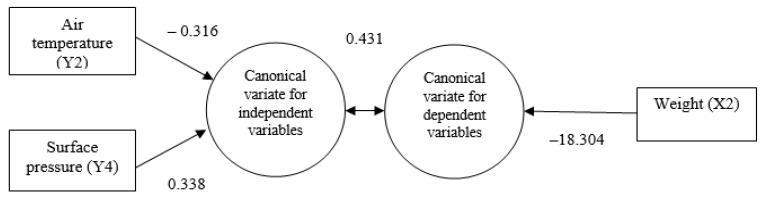
Canonical function 2 (adapted from Table 5).

Under the second canonical function, canonical loadings of explanatory variables, air temperature (−0.316), surface pressure (0.338), and the responsive or dependent variable, the weight (−18.304) of stinging catfish larvae, indicated a moderate canonical correlation (0.431%) between climatic variables and the growth parameters of stinging catfish larvae (Figure 5).

## 4. Discussion

The stinging catfish is a freshwater fish species with far-reaching farming potential. To improve stinging catfish farming, a comprehensive knowledge of embryonic and larval development is required. The embryonic and larval development of this fish is not fully understood, and only a few studies have been conducted [12,29,30] in this regard. This study was conducted to investigate and acquire detailed information on the embryonic and larval development of this commercially important fish in relation to climatic and water quality parameters.

### 4.1. Water Quality Parameters

The water quality parameters of this experimental study were within the acceptable limits with the temperature ranging from 27 to 31°C. The mean pH, TDS, and DO values were 8.06 ± 0.05, 344.27 ± 4.61 ppm, and 9.85 ± 0.53 mg/L, respectively. Water temperature at 28 ± 2.7 °C, pH at 7.4 ± 0.91, and DO at 5.91 ± 0.78 mg/L were recorded during induced breeding and embryonic growth of stinging catfish in a study [6]. In another study, the optimal range for temperature (29–31 °C), DO (5.0–6.0 mg/L), and pH (7.1–7.5) was reported for the spawning of stinging catfish [31]. An incubation temperature of 27.0–29.5 °C was also recorded for embryonic development of the Egyptian African catfish [32], which corresponds to the readings of this study.

### 4.2. Fertilization and Hatching Rates of Stinging Catfish

Fertilization rates (69%) and hatching rates (68%) of stinging catfish in this experiment were consistent to those reported in previous studies. According to a previous study, an approximately 98% fertilization rate of stinging catfish was recorded when pituitary gland extract (PGE) was injected at a dose of 75 mg/kg [28], which is higher than that in this study. Fertilization rates (70.45%) and hatching rates (75.33%) of stinging catfish using broodfish induced with human chorionic gonadotropin at 1000 IU/kg for both female and male fish, and with PGE at 6 mg/kg body weight for females and 2 mg/kg body weight for males, were reported in a study [33], which are almost similar to the present study. Such differences in the fertilization rate can be explained by differences in hormone types, dose variations, size of the broodfish, seasonal variations [34,35], environmental factors, and water quality parameters (alkalinity, DO, pH, and hardness) [36]. In a study, the fertilization and hatching rates for stinging catfish that were close to the results of this study while PGE was used [37]. In this study, the incubation period after fertilization using stinging catfish eggs at a water temperature of 29–31 °C was 22–23 h. Similar hatching times for stinging catfish ranging from 23 to 24 h at 29 ± 1.0 °C were recorded in another study [12]. A study shows that eggs hatched after 21–24 h of fertilization at around 22 °C [7] however, egg hatching can occur within 16–19 h at higher temperature (28–30 °C) [38]. Through this study, it has been proved that PGE injections can be used to breed stinging catfish at 27–30 °C for the easy production of fry as a result of high fertilization and hatching rates.

### 4.3. Embryonic Development of Stinging Catfish

The embryonic development of stinging catfish examined in this study occurred in hapas that were placed within a cistern. The results of this study are consistent with those of previous studies on embryonic development. According to previous studies, the size of fertilized eggs ranged between 1.3–1.4 mm [29], 1.3–1.5 mm [39], and 1.3–1.4 mm [40], which are consistent with this research findings of 1.35 mm. The size of the eggs might be associated with the environmental conditions and the size of the females collected from the wild [41]. It may also be related to individual parental investment and the food availability for the female fish to induce and spawn eggs. The fertilized eggs of stinging catfish became adhesive, similar to those of other catfish species, such as *Clarias batrachus*, *Mystus montanus*, and *Pangasius sutchi* [42,43,44]. The mode of cleavage observed in this study was similar to that of other catfish species, such as *Pangasius pangasius* [45]. In this study, the first and second cleavages occurred within 30–35 min and 40–50 min, respectively. The 8-, 16-, and 32-cell stages were observed at 60–70, 80–90, and 120–140 min post-fertilization, respectively. The first cleavage, 16-cell stage, and morula stage in stinging catfish within 30, 70-80, and 100 min after fertilization, respectively, were recorded in a study [29]. In another study, the first and second cleavages were recorded at 30 and 45 min, respectively [6]. In the same study, the 8-cell, 16-cell, and 32-cell stages were recorded at 70, 90, and 120 min post-fertilization, respectively [6]. In this study, the morula stage was attained between 2:50–3:00 h after fertilization. The blastula stage was attained approximately at 4:00–4:15 h after fertilization, and the gastrula stage was attained at 6:35–6:40 h post-fertilization, resulting in the formation of the germinal ring. A study reported that the morula was in progress at approximately 3:00 h post-fertilization, the blastula stage at 4:00 h post-fertilization, and that the gastrula stage was attained at 6:40 h of post-fertilization [6]. Another study showed that the morula formed at 3:07 h after fertilization, the blastodisc soon flattened into the blastula at 4:00 h after fertilization, and the gastrula formed at 7:06 h after fertilization [46]. It was reported that morula stages in stinging catfish were attained within 100 min post-fertilization, and recorded the gastrula stage at 7:00 h after fertilization [39]. The morula stage in *P. pangasius* is attained within 3:43 h, the blastula at 5:12 h and the gastrula at 7:27 h after fertilization [45]. In this study, the somatic stage gradually appeared after the gastrulation stage and proceeded to 22–25 somites, which resembled the observations of a study [40], where 18–20 somites in stinging catfish were recorded. In this study, twisting movement was observed at 1:00–1:50 h before hatching out of the embryo. The same findings were observed in a study [47], that is, twisting movement was observed at 1:00–2:00 h before hatching out of the embryos of stinging catfish. In another study [12], it was reported that the twisting movement of embryos was noticed before hatching. A similar hatching pattern was reported for stinging catfish [29] and was also observed for the other catfish species *Clarias batrachus* [41] and *Pangasius sutchi* [26]. In this study, the incubation period lasted for 23:00–24:00 h at a water temperature of 29–31 ºC, while an incubation period of 16:00–18:00 h for stinging catfish at a temperature of 26 °C was reported in another study [30]. Similarly, the required times for hatching of stinging catfish eggs were reported as 23:00–24:00 h and 20:00–24:00 h, respectively [40,48]. Temperature has a critical effect on the embryonic development of fish [17,49]. An incubation period of 16:00–18:00 h for stinging catfish was reported while the temperature remained at 26 °C [30]. The results related to embryonic development are consistent with those of many previous studies, as discussed above, which proves that the results obtained in this study can be used as a knowledge base to train hatchery owners.

### 4.4. Larval Development of Stinging Catfish

The growth performance of stinging catfish larvae was affected by the starter diet. In this experimental study, the average length and weight of the larvae in the hapa at 30th day after hatching were 25.38 ± 0.04 mm and 0.115 ± 0.056 g, respectively. The larvae were fed with egg yolk and powdered nursery feed containing 45% protein. Alongside these supplemental feeds, the larvae in the hapa had access to natural feed, such as zooplankton in the pond water. The better growth was observed in *Coregonus fera* fry fed with decapsulated artemia diets followed by egg yolk, brine shrimp, and German wean [50], which is consistent to the findings of this research. In another study, it was also reported that most fish larvae require live food at the onset of their exogenous feeding [51]. Additionally, the texture of the dry feed may have contributed to the poor development of fish larvae on inert diets (German wean), digestibility, and the leaching of nutrient in water, especially for egg yolk [52]. This could be because during the first several weeks of external feeding, the stomach was not functioning and there were not any proteolytic enzymes present [53], along with a lack of larval stimulation from the dry feed [54].

The mean length of newly hatched larvae of stinging catfish in this study was 2.78 mm. Findings showed that the mean length of newly hatched stinging catfish larvae was 2.5 mm [39], whereas the mean length of hatchlings for the same species was 2.72 mm [55]. In another study of *Channa striatus* species, the mean length of newly hatched fry was reported as 3.4 mm [47]. It is a known fact that the age and size of broodstocks affect the size of eggs and subsequently the size of fry [56]. In the investigation, the mouth pores of stinging catfish larvae were detected at 36:00 h after hatching, while in another study, the mouth openings in *H. longifils* appeared at 3:00–4:00 h after hatching [57]. Hatchling of stinging catfish commenced feeding after 72:00 h in this study, where very few larvae started exogenous feeding on the 3rd day, with the majority starting external feeding on the 4th day in another study [58]. It was also observed that newly hatched larvae of *Pangasius sutchi* vigorously took supplementary feed within 3–6 days of hatching in a different study [44]. In this study, complete yolk sac absorption was observed on the 6th day, when the stinging catfish larvae gained an average length of 6.44 ± 0.06 mm, while the yolk sac of *Pangasius sutchi* larvae was fairly well absorbed by 3 days after hatching [44]. The yolk sac of *M. gulio* larvae was depleted on the 3rd day after hatching, at which time the larvae measured 3.86 mm in total length [59]. The yolk sacs of *Clarias gariepinus* were reported to be completely absorbed at about 60:00–66:00 h after hatching at a water temperature range of 25–28 °C [32,60]. 

### 4.5. Correlation between Larval Development and Climatic and Water Quality Parameters

According to canonical correlation analysis, there was a strong correlation (79%) between water quality parameters and growth parameters, explained by the first function, whereas a moderate correlation (43%) existed for the second correlation between climatic variables and growth parameters. The contributions of all parameters under climatic variables, water quality parameters, and growth parameters are shown as individual percentages of variance alongside the collective loading percentage of variance. Under the first canonical correlation, pH, DO, and weight showed a positive correlation, as high loading variance implies that larval development significantly depends on pH and DO. This finding is consistent with a previous study [61] reporting that environmental variables, such as DO and pH, strongly influence fish species during embryonic and larval development. Moreover, pH is highly relevant to the survival and development of eggs and fish larvae. Water pollution, agricultural runoff, and increased CO_2_ resulting from climate change lead to the extensive mortality of fish larvae [62,63,64]. In this sense, studies focusing on the effects of low pH in the early stages of fish development, such as increased incubation time, unviability of eggs, mortality, and larval deformities, have already been conducted [22,65]. Moreover, low pH triggers degeneration of gill tissue and increases mucus secretion, which can kill fish [66].

In contrast, reduced amounts of DO can affect the growth and development of eggs, as well as the swimming, eating, and reproductive abilities of juveniles [67]. Hypoxia has been demonstrated to delay embryonic growth and hatching in several fish species [68]. Under hypoxia, fish embryos lose their usual synchronization, and a lack of pigmentation along with defects in spinal and vascular development are prevalent [68]. Fertilized eggs take a comparatively large amount of time (96:00–260:00 h) to hatch under hypoxia, with only 4.9% hatching and the remainder dying [69].

The bivariate correlation matrix indicated a significant positive correlation (0.697) between water temperature and air temperature. Water temperature influenced embryonic and larval development, as air temperature and weight in the second canonical function contributed significantly among other variables with moderate loadings, and surface pressure showed a positive correlation with a moderate loading of variance. This explains why temperature and surface pressure had a significant impact on embryonic and larval development. Embryos and larvae are life stages in fish that are highly thermally sensitive. High temperatures accelerate the rate of development, resulting in the appearance of certain structures at smaller larval sizes [70], as has been observed for fin formation and metamorphosis [71,72]. High temperature during embryogenesis induced early hatching, mouth opening, and advanced skeletal ossification at 16 days after hatching in *Atractosteus tropicus* [73]. Long-lasting effects of early temperature exposure have been identified in metamorphosing gilthead seabreams, including effects such as decreased critical swimming speed and the incidence of caudal fin abnormalities [74].

## 5. Conclusions

Embryological studies are important for successful and profitable breeding programs, and are indispensable tools, particularly for quality fish seed production for commercial aquaculture. The current study contributes to an advanced knowledge on the identification and characteristics of the morphological developmental stages of stinging catfish larvae, which will play an important role in the preparation of hatchery management manuals for hatchery operators. Through this research, the various stages of embryonic development are concisely presented with microphotographic images, which will make hatchery management simple, thereby increasing hatchery productivity manifold. Larval and post-larval developmental stages are very well characterized and well described. Finally, canonical correlation analysis was applied to provide detailed statistics on how climatic and water quality parameters were related to larval growth. The knowledge gained through this study of various stages of embryonic and larval development of stinging catfish using photomicrographic methods, image analysis, and the interrelationship between larval growth and climatic and water quality parameters will play an important role in hatchery operations and hatchery productivity in Bangladesh.

## Figures and Tables

**Table 1:** Climatic variables and water quality parameters.

Parameter	Highest	Lowest	Average ± SE
Air temperature (°C)	31.37	26.60	28.87 ± 0.23
Surface pressure (kPa)	100.62	99.97	100.36 ± 0.03
Relative humidity (%)	93.44	71.19	81.87 ± 1.17
Water temperature (°C)	31.00	27.00	30.46 ± 0.18
pH	8.60	7.40	8.05 ± 0.05
DO (mg/L)	11.81	4.90	9.85 ± 0.53
TDS	178.00	148.00	161.53 ± 1.41
Ammonia (mg/L)	0.13	0.13	0.13

**Table 2:** Different stages of embryonic development of stinging catfish in different time period.

Figure No.	Development Stages	Size in Diameter (μm)	Development Time Range in Both System (Hour: Minutes)	Characteristics
A	Fertilized egg	1355.06 ± 17.15	00:00	Round, transparent and adhesive in nature.
B	Blastodisc formation	1364.88 ± 28.43	00:20–00:25	Cytoplasm accumulated at the anterior part to form animal pole or blastodisc where cell divisions occur. Reddish blastodisc on the pole of fertilized eggs were easily identified with the naked eye.
C	2-cell	1438.09 ± 25.29	00:30–00:35	Two cells over the yolk sphere were clearly visible at the first cleavage stage.
D	4-cell	1440.59 ± 47.69	00:40–00:50	Four cells at the animal pole produced by second cleavage.
E	8-cell	1457.19 ± 9.92	1:00–1:10	Third cleavage produced eight cells arranged in two rows of four cells where little overlapping of blastomeres was observed.
F	16-cell	1472.02 ± 13.17	1:20–1:30	Sixteen cells were produced in fourth cleavage. At this stage cell counting becomes difficult and cell size becomes reduced due to successive cell division.
G	32-cell	1472.16 ± 23.46	2:00–2:20	Fifth cleavage where the blastomeres were visible in 2-3 layers producing 32 cells.
H	64-cell	1475.81 ± 19.64	2:30–2:40	Sixth cleavage where overlapping of blastomeres was observed producing 64 cells and were placed in 2–3 layers.
I	Morula	1480.44 ± 30.88	2:50–3:00	Repeated cell divisions leading to the formation of multicellular blastodisc where the cells were very small and gave a flowery look at the animal pole.
J	Blastula	1486.01 ± 1.22	4:00–4:15	Epiboly formed as embryonic shield on the animal pole and blastoderm was compressed occupying more than half of the area over the yolk sphere.
K	Gastrula	1491.03 ± 30.56	6:35–6:40	Germinal ring was formed with two somites where thick layer of blastoderm occupied 3/4 area over the yolk sphere. The broader end became the future cephalic part of the embryo.
L	Somatic formation	1509.26 ± 40.58	9:00–18:00	Antero-posterior axis become distinguishable, cephalic portion become broader, and embryonic rudiment became distinct with two somites. Further development of somites reached 22–25, and the yolk became completely encircled by kidney shaped embryo with clear distinction of head and tail.
M	Yolk plug	1523.80 ± 31.66	19:00–19:30	Yolk plugs are the remaining patch of endodermal cells formed and exposed on the vegetal surface of the blastula.
N	Twisting movement	1551.90 ± 23.59	20:00–21:00	Tail became free from yolk sphere and frequent embryonic twitching movements occurred as the embryo tried to rupture the perivitelline membrane.
O	Pre-hatching	1647.44 ± 40.61	21:00–22:00	Wriggling movement increased as chorion wall still enclosed the embryo, heartbeat increased to 68 times per minute.
P	Newly hatched larvae	2780.08 ± 43.67	22:30–23:00	The egg membrane was broken down and the embryo tail first emerged, followed by the trunk and head region. It took around 2 h for completion of the hatching from twisting movement of the embryo.

**Table 3:** Detailed information of larval development of stinging catfish in the hapa.

Stage of Larvae	Length (mm)	Characteristics
Hatchling	2.78	Mean length of the newly hatched larvae was 2.78 ± 0.04 mm, body color was transparent to brownish. Body was laterally compressed and head was attached to yolk sac looking a little bent at the anterior portion. Eyes were unpigmented, mouth or mouth cleft was not distinguishable. The pale greenish yolk sac was oval in shape with the diameter of 1.28 ± 0.03 mm. A thin and transparent fin fold surrounded the caudal region which extended up to the yolk sac (Figure 3a). A functional heart was noticed with the heartbeat of 180 times per minute.
4-h old larvae	2.92	The 4 h old larvae were brownish in color and, measured about 2.92 ± 0.02 mm in length. The anal pore position was almost at the mid ventral point and was not opened. The optical cups were visible, but the eyes were unpigmented. The two chambered heart became more distinct with circulation of body fluid around the notochord, brain, and yolk. A tube-like digestive tract was visible which emerged from the posterior-dorsal side of the yolk sac. Barbels were not yet developed, and reddish blood corpuscles represented formation of hemoglobin. Scattered melanophores were observed on the yolk sac and on the unpaired fin.
8-h old larvae	3.07	The average length of 8 h old larvae was about 3.07 ± 0.01 mm. The yolk sac became elongated and some melanophores appeared on the head region, ventral, and dorsal side of the body. Heart and brain of the larvae were distinctly visible. Some pigments were visible on the iris. The larvae became very active and light sensitive at this stage.
24-h old larvae	3.13	The 24 h old larvae became 3.13 ± 0.33 mm in its average length. The yolk sac reduced and dark pigmented eyespot appeared on the anterior part of the head. The upper and lower jaws were formed, and alimentary tract was distinct. The mouth and the anal openings were still closed, and the heart was clearly visible in front of the yolk. The blood circulatory system was fully functional. Melanophores were scattered on the dorsal fin fold and trunk region.
36-h old larvae	4.36	The average length of 36 h old larva attained 4.36 ± 0.3 mm. The eyes were spherical in shape with dark pigmentation. Yolk sac was further reduced. Preanal and postanal length were 2.15 mm and 2.21 mm, respectively. Four pairs of tiny barbels appeared (one pair = maxillary, one pair = nasal, two pair = mandibular). The mouth and the vent just opened (Figure 3c).
48-h old larvae	4.57	The larvae attained 4.57 ± 0.14 mm in length with a postanal length of 2.29 mm and preanal length of 2.28 mm. The dark and prominent eyeball diameter was 232.95 µm. The barbels became elongated and the yolk reserve was further reduced in size. The anal aperture and opercula were distinct. Denser melanophores were visible at the head region compared to the body. The pouch-like stomach and the alimentary canal became distinct. Blood circulation was noticed in the opercula, head, and tail region.
3-d old larvae	5.54	The average length of the 3 d larvae reached 5.54 ± 0.29 mm with a preanal and postanal length of 2.68 mm and 2.86 mm, respectively. The body became brownish in color and the mouth and anus become fully functional. The head was prominent and head length reached 1.33 mm. The nasal, maxillary, and mandibular barbels became 0.817 mm, 1.4 mm, and 1.18 mm in length, respectively. Body pigments were more concentrated in the anterior region, the yolk material was reduced in size.
6-d old larvae	6.44	The 6 d old larval average length reached at 6.44 ± 0.06 mm and weight 4.3 ± 0.3 mg. The yolk material became completely absorved, body color became brownish black and caudal fin rays were clearly noticeable with eight rays. Eyeballs were large and barbels become more elongated.
10-d old larvae	7.36	The average length of 10 d old larvae was 7.36 ± 0.43 mm and weight 6.3 ± 0.9 mg with a preanal and postanal length of 3.30 mm and 4.06 mm, respectively. Dorsal and anal fins were almost separated from the caudal fin, and the caudal fin was clearly seen with eight fin rays. The larvae started frequent surfacing movements and active swimming.
15-d old larvae	8.73	The average length of 15 d old larvae reached at 8.73 ± 0.48 mm with weight 15.0 ± 3.1 mg, preanal length 3.67 mm and post anal length of 5.06 mm. A total of eight caudal fin rays became distinguished, dorsal fin formed, spine of the pectoral fin became distinguished. Due to numerous pigments the body, color of the larvae became opaque.
20-d old post-larvae	11.12	The average length of 20 d old post-larvae reached 11.12 ± 0.28 mm with average weight 22 ± 2.1 mg. Body color of the larvae became darker due to huge pigmentation at this stage. Barbels became more elongated.
25-d old post-larvae	17.39	The mean length was observed 17.39 ± 0.95 mm and the body weight was about 36.30 ± 2.9 mg. The body color of the fish became dark reddish brown at this stage.
30-d old post-larvae		The average length and weight of post-larvae were recorded 32.00 ± 2.00 mm and 115 ± 56.3 mg, respectively. The fish became elongated and was observed to be like an adult with all morphological characters. On the head of the fish, two depressions were formed. The terminal mouth was transverse and wide.

**Table 4:** Pearson correlation between key climatic and water quality parameters.

	Air Temperature	Surface Pressure	Humidity	Water Temperature	DO	pH	TDS
Air Temperature	1						
Surface Pressure	−0.123	1					
Humidity	−0.918 **	0.005	1				
Water Temperature	0.697 **	−0.264	−0.511 **	1			
DO	−0.401 *	0.283	0.595 **	−0.045	1		
pH	−0.268	0.139	0.398 *	−0.102	0.642 **	1	−0.233
TDS	0.453 *	−0.203	−0.585 **	0.116	−0.605 **	−0.233	1

**. Correlation is significant at the 0.01 level (2-tailed). *. Correlation is significant at the 0.05 level (2-tailed).

**Table 5:** Canonical structure loading for association between climatic and water quality parameters, and growth parameters of stinging catfish.

Variables No.	Variables	Canonical Function	
		1	2
	Independent Variables		
Y1	Water Temperature	0.003	0.021
Y2	Air Temperature	−0.071	−0.316
Y3	Humidity	−0.042	−0.071
Y4	Surface Pressure	−0.023	0.338
Y5	pH	0.321	0.220
Y6	TDS	−0.006	−0.001
Y7	DO	0.265	0.036
	Dependent Variables		
X1	Length	−0.058	0.069
X2	Weight	6.607	−18.304
	Canonical Correlation	0.791	0.431

## Data Availability

The data presented in this study are available on request from the corresponding author.
